# Artificial intelligence (AI)-driven technologies for managing pediatric speech and language therapy: A scoping review

**DOI:** 10.1177/20552076251376533

**Published:** 2025-11-05

**Authors:** Milad Dadgar, Cathy Ennis, Kesego Mokgosi, Robert Ross

**Affiliations:** 1 School of Computer Science, 8819 Technological University Dublin, Ireland; 2 School of Computer Science, 8798 Maynooth University, Ireland

**Keywords:** Automated speech therapy, speech sound disorder, automatic speech recognition, audio processing, machine learning

## Abstract

**Objective:**

Despite the high demand for speech and language therapy (SLT) for children with speech sound disorders (SSDs), accessible services remain limited. Technology-driven efforts have led to the development of systems and applications to assist children, parents, and therapists in the SLT process. AI and machine learning (ML), particularly through automatic speech recognition and audio processing techniques, play a central role in these advancements. This scoping review examines studies focusing on these techniques for managing the SLT process.

**Methods:**

To include the most relevant studies, a systematic search was conducted on 3 February 2025 across five major databases (PubMed, Scopus, ScienceDirect, ACM Digital Library, and IEEE Xplore), following the Preferred Reporting Items for Systematic Reviews and Meta-Analyses for Scoping Reviews guidelines. After applying our criteria, 30 of the 188 identified studies met the eligibility requirements.

**Results:**

These studies predominantly utilize deep neural networks, ML classifiers, acoustic features, and audio processing techniques to detect SSDs. The findings demonstrate the effectiveness of these applications to support therapists in diagnostics. Moreover, computer-based tools have proven more engaging for children than traditional therapy by offering personalized therapy plans and real-time feedback. These systems enable therapists to monitor progress and adjust treatments.

**Conclusion:**

This review provides an overview of AI-assisted SLT models, highlights gaps, and suggests directions for future research. It shows the effectiveness and potential of AI in enhancing the SLT process. However, challenges related to data privacy, accessibility, and the need for clinical validation persist and need to be addressed in the future.

## Introduction

Effective communication is a key determinant of success in modern life, and speech is the primary mode of communication we learn from early childhood.^1^ However, some children face challenges in developing clear speech, which can negatively affect their social interactions and future opportunities. Speech sound disorders (SSDs) encompass a range of difficulties in speech production and may be associated with underlying conditions such as structural anomalies (e.g. cleft palate), neurodevelopmental disorders (e.g. autism spectrum disorders, cerebral palsy, and Down syndrome), phonological disorders which are language-specific, and motor impairments, all of which can impact the clarity and accuracy of speech sounds.^2,3^ Early and adequate diagnosis of speech disorders can contribute to the quality of treatment and thus to treatment success rates.^4^ As reported by Niegia et al.,^5^ up to 24.6% of children are affected by some form of SSD. However, only a few receive appropriate and consistent treatment. Speech and language therapy (SLT) is widely recognized as an effective intervention for managing SSDs.^6^ However, despite the growing demand for SLT services, access remains limited due to a shortage of qualified therapists and available appointments.^7^ Studies indicate that personalized and consistent therapy plans are critical for effective treatment,^8^ but many children lack access to these tailored services.^7^ Personalized SLT plans require significant time and effort from therapists to account for the specific needs of each child, which can be resource intensive.^9^ In addition, therapy sessions must be engaging for the children to ensure that they adhere to prescribed exercises.^10^ As a result, the high cost, long waiting times, and inaccessibility of traditional SLT approaches pose significant challenges to providing comprehensive care for children with SSDs.

In today’s technology-driven world, computer systems, mobile applications, and interactive games have emerged as promising tools in addressing the challenges in SLT. These digital solutions, combined with advancements in artificial intelligence (AI) and machine learning (ML), offer scalable approaches to diagnosing and managing SSDs. Specifically, automatic speech recognition (ASR) systems and natural language processing technologies, coupled with signal processing techniques, can transcribe speech and analyze audio and acoustic features to detect impairments. By incorporating these approaches into SLT applications, these tools can provide SSD detection models, real-time feedback, track progress, and engage children through interactive games, making therapy more accessible, effective, and engaging. Such technologies have shown potential in addressing the shortage of therapists and reducing the time and cost associated with traditional therapy.^11^

Although there is significant research focused on AI-driven systems for SLT, relatively few studies address the unique challenges posed by children’s speech. Children’s speech varies significantly across individuals and evolves rapidly, making it difficult for AI models, particularly those trained on adult data, to accurately analyze and detect impairments in younger users.^12^ Additionally, the lack of large, comprehensive datasets on children’s speech, especially for those with SSDs, presents a major obstacle for developing robust AI models. Data privacy concerns also limit the availability of extensive corpora, further complicating the task of refining AI systems for pediatric speech therapy.

The same holds true for survey papers on SLT systems for children. While various efforts have been made to consolidate information and research in review papers, few are specifically tailored to children’s speech therapy. Targeted reviews for children’s speech are essential due to the unique characteristics of children’s speech, for example, child speech exhibits greater inter- and intra-speaker variability compared to adult speech, posing additional challenges for processing and analysis.^12^ This variability arises due to factors such as developmental differences in articulation, inconsistent speech patterns, and limited phonemic inventories.^13^ Although there are valuable existing reviews for pediatric SLT, they primarily focus on applications and games, emphasizing game design and high-level system analysis.^14,15^ However, a deep understanding of the current AI models and ML algorithms is necessary for future researchers aiming to build on current approaches. For instance, Atwell et al.^14^ briefly list the ML algorithms utilized in its included studies without providing a detailed analysis about their development process, evaluation, and the results. Similarly, Saeedi et al.^15^ primarily focused on game design rather than on AI models or implementation. In Moulaei et al.,^16^ tele-speech therapy is evaluated from a high-level perspective, incorporating relevant studies. While the paper primarily examines the advantages and practicality of these systems, it does not delve into AI-driven solutions or the in-depth development aspects of these technologies. These gaps in detailed evaluation of AI and ML methods specific to children across existing studies highlight the need for a scoping review to systematically map the current landscape, identify key characteristics of ML-based SLT systems, and uncover areas requiring further in-depth research. In light of this, in this review, we provide a detailed analysis and explanation of the ML centric systems currently developed for pediatric SLT, showcasing state-of-the-art technologies to serve as a foundation for future research. Additionally, we categorize these works by factors such as ML model architectures, application features, and more. These categorizations offer a structured overview that can serve as a valuable reference for examining the current state of SLT technologies and identifying existing gaps in the field.

Given these gaps in the literature and the growing importance of AI-driven tools, our review covers works that utilize speech and audio data for SSD detection, focusing on the AI techniques and data processing methods employed in these applications. By analyzing the models used, including deep neural networks (DNNs) and ML classifiers, we identify key trends and challenges in the field. A consistent challenge highlighted across nearly all the reviewed studies is the scarcity of child speech data. This limitation applies to both typically developing (TD) children and children diagnosed with SSD, complicating the training and evaluation of these models. We also discuss the effectiveness of real-time feedback, the quality of the acoustic features employed, and the contributions of these systems to the broader goal of creating reliable, fully automated speech therapy tools for children.

## Methodology

The main goal of this scoping review is to gather and analyze all relevant works in the field of pediatric SLT, focusing on their technical models and technologies. While some papers detail their implementation methods, others primarily discuss results and final products. We aimed to extract the most pertinent information from the current literature, considering relevance, time constraints, and effectiveness.

### Scope

At the outset, we formulated six key research questions (RQs) to guide this review, which will be addressed in the results section:

- **RQ1:** What are the technical goals of these papers?
- **RQ2:** What languages and participant selection criteria are used in these studies? (This term refers to both the children whose data was utilized for development of the models and those who participated in the experiments.)
- **RQ3:** What pathologies are targeted by these studies?
- **RQ4:** What are the application features provided by these systems?
- **RQ5:** What are the AI models and speech properties utilized by these studies?
- **RQ6:** What are the datasets utilized or created by these studies?

In this review, we aim to briefly identify the primary technical goals of the systems implemented in the included studies. While some studies focus on the detection and classification of SSDs, others concentrate on developing serious games and applications to engage children in practice and rehabilitation. This analysis aligns with the objectives outlined in RQ1.

Based on RQ2, we will investigate the spoken languages targeted by the studies, as language-specific nuances can greatly impact the effectiveness of speech therapy interventions and the development of automatic systems for SSD detection and treatment.^17^ In addition, we are going to take a look at the profile of participants within these studies.

RQ3 examines the different types of SSDs that these systems address. Since each SSD type presents unique symptoms, most implemented systems are specifically trained for and limited to a particular pathology. Therefore, it is important to analyze the solutions corresponding to each type of pathology.

RQ4 focuses on the application features provided by these systems. Key features analyzed include disorder detection capabilities, real-time feedback, clinical report generation, and the presence of any interactive or game-based elements.

Regarding RQ5, we will explore technical aspects of these systems. Specifically, we will examine their classification models and algorithms, the extent to which they utilize ASR techniques, the audio properties they extracted and utilized to feed to their model, and other AI-related technologies employed. Additionally, we will discuss various challenges developers encounter when dealing with child speech.

Finally, RQ6 investigates one of the core elements in this field: data. As data forms the foundation of any reliable ML model, we will examine the datasets used or custom-built in these studies, with attention to how they were adapted to meet specific research needs. This analysis aims to provide future researchers with valuable resources for developing effective models.

### Search terms

To find the most relevant studies, we conducted a comprehensive search across five major databases: PubMed, Scopus, ACM Digital Library, ScienceDirect, and IEEE Xplore. The search, performed on 3 February 2025, focused on three main groups of search terms applied to the title, keywords, and abstract fields. The initial search yielded over 11,000 papers; to refine the results and ensure a more robust and replicable approach, we further refined the candidate set with a subsequent search, which was applied to titles only. Within each search term group, the OR operator was used to include all relevant variations, and the three groups were then combined using the AND operator to capture studies that matched across all categories. The complete set of search terms is presented in Table 1.

**Table 1.** Search terms used.

Groups	Keywords
Group 1 (technology-related terms)	Tool, Information System, Software, System, Computer, AI, Artificial Intelligence, Online, Video Game, Machine Learning, Application, Mobile, Gamification, Automatic, Automated, ML, Technology, Dataset, Database, Data Collection
Group 2 (speech-language therapy terms)	Speech Therapy, Language Therapy, Speech Disorder, Sound Disorder, Voice Disorder, Speech Language Therapy, SSD, SLT, Language Disorder, Speech Pathology, Stuttering, Speech Rehabilitation, Cleft Palate, Articulation Disorder, Phonological Impairment, Apraxia, Dysarthria, Aphasia, Dysphonia, Phonological Disorder, Dyspraxia, Speech Delay, Speech Impairment, Language Impairment
Group 3 (children-related terms)	Children, Child, Preschool, School, Young

### Inclusion and exclusion criteria used

The refined search yielded 188 papers with 64 duplicates. After removing duplicates, the inclusion and exclusion criteria were applied. Table 2 presents the criteria utilized in this study.

**Table 2.** Inclusion and exclusion criteria.

Inclusion criteria	Exclusion criteria
Papers focused on audio or video data from users.	Papers without audio or video processing technologies.
Papers on creating databases for SLT.	Papers that do not detail their technical methods.
Papers utilizing AI, ML, and DL techniques on data recorded from low-cost devices.	Papers focused solely on evaluation without introducing new approaches.
Full-text must be available.	Papers that do not involve interaction with users or user-centric data.
Papers published from 2018 onwards.	Papers lacking interventional goals.
	Non-peer-reviewed papers.

SLT: speech and language therapy; AI: artificial intelligence; ML: machine learning; DL: deep learning.

**Figure 1.**
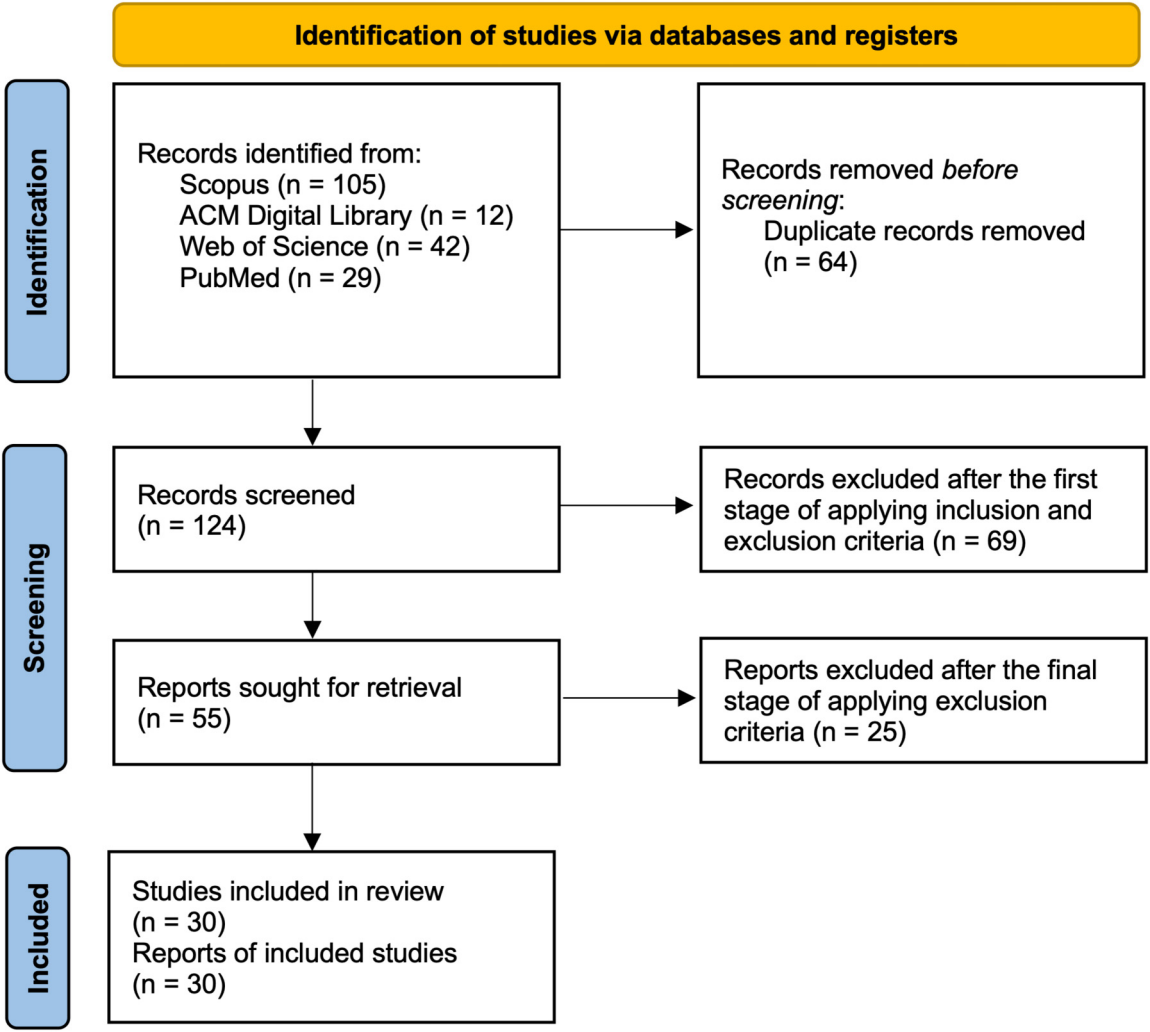
Preferred Reporting Items for Systematic Reviews and Meta-Analyses (PRISMA) flow diagram illustrating the paper selection process.

In the first stage of the full-text review, 55 papers were selected based on our inclusion criteria. In the second stage, a more thorough screening was conducted, applying the exclusion criteria carefully and in detail. As a result, 30 papers met all the criteria and were retained. These papers form the basis of the analysis in the following sections. The selection process for papers and data extraction and analysis follows Preferred Reporting Items for Systematic Reviews and Meta-Analyses for Scoping Reviews^18^ roles, which we diagram in Figure 1. This scoping review was not registered in any review registry.

Data from the included studies were systematically charted using a spreadsheet specifically designed for this review. Each row represented an individual paper, and columns corresponded to key extracted variables, such as author, year, country, study aims, methods, results, and conclusions. Due to the iterative nature of the collaborative evaluation of extracted data among team members and the review process, the spreadsheet was continuously shared and updated, enabling all reviewers to verify, refine, and confirm the accuracy and completeness of the charted information.

**Figure 2.**
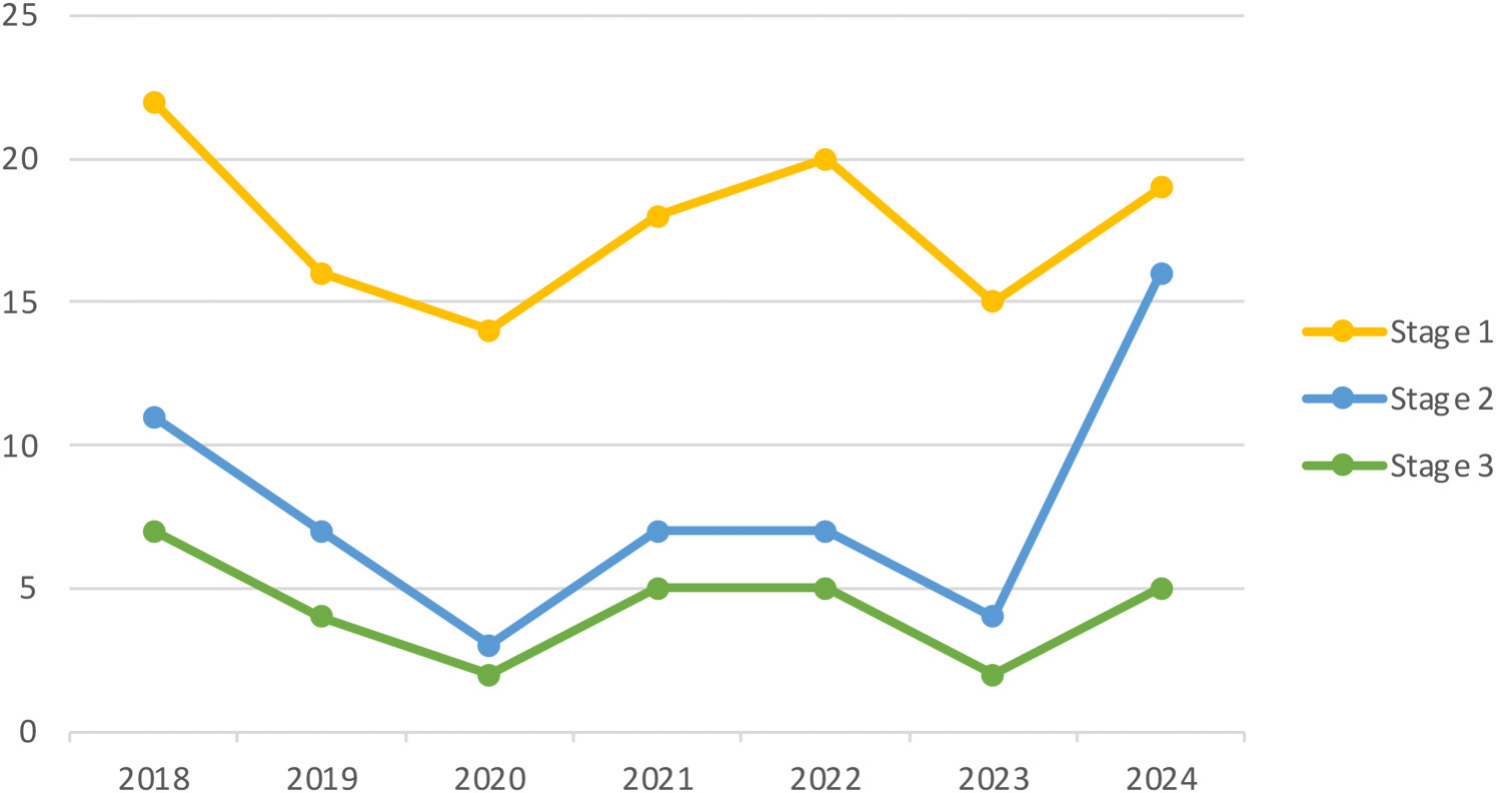
The publication years of the included studies. Stage 1 represents the initial count of papers identified by our search terms, following the removal of duplicates. Stage 2 indicates the number of papers retained after an initial screening, which excluded those that were not relevant. Stage 3 reflects the final count of studies included after a comprehensive review and application of the exclusion criteria.

Furthermore, Figure 2 presents the distribution of publication years for the included papers broken down by their inclusion or exclusion.

## Results

Table 3 presents a brief overview of all papers that were retained for full analysis. These papers are categorized according to their technical goal, country or origin and language, pathology, and year of publication.

**Table 3.** Overview of the included studies.

Ref	Title of article	Year	Country/Language	Pathology	Technical goal
A	Automatic children speech sound disorder detection with age and speaker bias mitigation^23^	2024	KOR/KOR	–	SSD detection
B	Automatic speech recognition (ASR) for the diagnosis of pronunciation of speech sound disorders in Korean children^24^	2024	KOR/KOR	–	SSD detection
C	Automatic detection of speech sound disorder in Cantonese-speaking pre-school children^25^	2024	HKG/YUE	–	SSD detection
D	Using artificial intelligence for assessment of velopharyngeal competence in children born with cleft palate with or without cleft lip^26^	2024	SWE/SWE	Cleft palate	SSD detection
E	Computer-assisted syllable analysis of continuous speech as a measure of child speech disorder^27^	2024	USA/ENG	–	SSD detection
F	“Phrasefluent’—an automated solution for children’s speech disorders identification and therapy treatment^28^	2023	QAT/ENG	Stuttering	SSD detection
G	A study on using duration and formant features in automatic detection of speech sound disorder in children^29^	2023	HKG/YUE	–	SSD detection
H	wav2vec2-based speech rating system for children with speech sound disorder^30^	2022	SWE/SWE	–	SSD detection
I	Feature engineering and machine learning for computer-assisted screening of children with speech disorders^31^	2022	USA/ENG	–	SSD detection
J	Identifying language disorder in bilingual children using automatic speech recognition^32^	2022	USA/ENG & SPA	–	SSD detection
K	Automatic detection of speech sound disorder in child speech using posterior-based speaker representations^33^	2022	HKG/YUE	–	SSD detection
L	The SLT 2021 children speech recognition challenge: Open datasets, rules and baselines^34^	2021	CHN/CMN	–	SSD detection
M	Artificial intelligence for dysarthria assessment in children with ataxia: A hierarchical approach^35^	2021	ITA/ITA	Ataxia	SSD detection
N	The automatic detection of speech disorders in children: Challenges, opportunities, and preliminary results^12^	2020	QAT/ENG	–	SSD detection
O	Toward explainable automatic classification of children’s speech disorders^4^	2020	ISR/HEB	Apraxia	SSD detection
P	Automatic screening of children with speech sound disorders using paralinguistic features^36^	2019	AUS/ENG	–	SSD detection
Q	Improving ASR systems for children with autism and language impairment using domain-focused DNN transfer techniques^37^	2019	USA/ENG	Autism spectrum disorder	SSD detection
R	Automatic classification possibilities of the voices of children with dysphonia^38^	2018	HUN/ENG	Dysphonia	SSD detection
S	Automatic analysis of pronunciations for children with speech sound disorders^21^	2018	USA/ENG	–	SSD detection
T	An automated assessment tool for child speech disorders^39^	2018	HKG/YUE	–	SSD detection
U	Blending situation awareness with machine learning to identify children’s speech disorders^40^	2018	BRA/POR	–	SSD detection
V	Research on the application of artificial intelligence in the rehabilitation training of children with speech disorders^41^	2022	CHN/ENG	–	SLT game
W	SATReLO: A tool to support language therapies for children with hearing disabilities using video games^42^	2021	ESP/SPA	–	SLT Game
X	Customizable serious speech therapy games with dynamic difficulty adjustment for children with sigmatism^43^	2021	PRT/POR	Sigmatism	SLT Game
Y	Sustained vowel game: A computer therapy game for children with dysphonia^44^	2019	PRT/POR	Dysphonia	SLT Game
Z	Design of a web tool to support language therapy for children with cleft lip and/or palate^45^	2019	MEX/SPA	–	SLT Game
AA	SLT-Game: Support system for therapies of children with communication disorders^46^	2018	ECU/ENG	–	SLT Game
AB	Assistive mobile app for children with hearing & speech impairment using character and speech recognition^47^	2018	PHL/ENG	–	SLT Game
AC	Tingog: Reading and speech application for children with repaired cleft palate^48^	2018	PHL/ENG	Repaired cleft palate	SLT Game
AD	A system to support children in speech therapies at home^49^	2021	ITA/ITA	–	SLT Game

Country/language are given as three-letter ISO codes. (SSD: speech sound disorder; SLT: speech and language therapy.)

### Technical goal

We categorized the primary technical goals of the included studies into two main groups: SSD detection and SLT games. The first category includes papers A, B, C, D, E, F, G, H, I, J, K, L, M, N, O, P, Q, R, S, T, and U, with the primary goal of detecting impairments based on speech input. Some studies target specific conditions, such as Paper A, which focuses on stuttering and articulation disorders, Paper H, which addresses apraxia, Paper K, which concentrates on autism spectrum disorder, Paper L, which is focused on dysphonia, and Paper D focuses on repaired cleft palate. Other studies in this category work on SSD detection generally. The second category comprises papers V, W, X, Y, Z, AA, AB, AC, and AD, which focus on developing engaging games designed to attract children to practice and actively participate in their therapy plans. The technical goals of each paper are summarized in Table 3.

### Language and participants

SLT systems are inherently dependent on the language they are trained in, which poses a limitation, as no current multilingual system can accommodate children across different languages universally. Given this, it is essential to examine the languages covered in these studies. Most of the literature focuses on a single language—primarily English—with some studies addressing other languages such as Spanish, Italian, Portuguese, Cantonese, and Korean. Study J, however, covers both English and Spanish. Table 3 presents the languages covered in the reviewed studies.

As previously mentioned, this review focuses on systems developed for children, making age a key criterion for participant selection. Overall, the studies cover an age range of 2 to 16 years, with an average focus on children between 4 and 10 years old. The number of participants varies widely across studies, with the smallest sample sizes of 12 in paper W and 14 in paper Y, and the largest sample sizes of 1362 in paper U and 709 in paper A. Gender distribution is rarely specified; however, papers J, O, P, Q, R, S, and Y report a total of 231 (63%) boys and 137 (37%) girls. A noteworthy finding about pediatric SSDs is the significant gender disparity in their prevalence. Studies demonstrate that boys are more likely to be diagnosed with SSDs compared to girls, with a meaningful male-to-female ratio of approximately 2:1 .^19^ This pattern has been consistently observed in research, which highlights the higher vulnerability of boys to persistent SSDs. Additionally, according to papers A, C, E, F, G, H, I, J, K, M, P, Q, R, S, and Y, the test samples include 1824 (64%) TD children and 1044 (36%) children with SSD. Table 4 provides a summary of the participants’ characteristics. While most of the included studies do not provide detailed information about participants, we present the characteristics of those mentioned where available.

**Table 4.** The summary of participants’ characteristics.

Paper	Number	Age range (years)	Males	Females	SSD^1^	TD^2^
A	709	2 to 10	–	–	286	423
B	134	3 to 7	–	–	–	–
C	578	3 to 6	–	–	150	428
D	60	3 to 5	–	–	44	16
F	50	4 to 12	–	–	30	20
G	578	3 to 6	–	–	150	428
H	28	4 to 10	–	–	16	12
I	51	3 to 8	–	–	12	39
J	84	–	46	38	25	59
K	415	3 to 6	–	–	150	265
M	55	–	–	–	37	18
O	24	4 to 16	18	6	–	–
P	56	6 to 11	44	12	28	28
Q	45	6 to 9	36	9	45	0
R	59	5 to 10	39	20	25	34
S	86	4 to 12	42	44	43	43
U	1362	3 to 8	–	–	–	–
W	12	6 to 12	–	–	–	–
Y	14	4 to 5	6	8	3	11
**Overall**	4400	2 to 16	231	137	1044	1824
**Mean**	232	4 to 10	33	20	70	122
**Std**	342.87	–	13.99	14.26	76.23	164.06

1 Children with speech sound disorders.

2 Typically developing children.

### Pathology

Different types of SSDs have distinct symptoms and require tailored rehabilitation processes. For example, dysphonia and stuttering, among other SSDs, are pathologies with unique symptoms, emphasizing the need to understand which specific impairments these models target and are trained for. While most studies focus broadly on SSD detection without targeting specific types, some address particular impairments and classify specific disorders. Table 3 lists the pathologies addressed by these studies. Notably, almost all the papers concentrate on children with SSD, with only one study [AB] including both children and adults.

### Application features

The selected papers employ various approaches to address gaps in the literature and enhance SLT systems. There are multiple components involved in making an SLT system. Some studies focus on developing engaging games designed to motivate children with SSD to follow through on treatment plans and complete their practices. Others utilize AI techniques to introduce new models that accurately detect or classify speech impairments. Additionally, some works explore innovative techniques for data collection and pre-processing to maximize efficiency. In this section, we are going to cover the most common application features of these systems.

Disorder detection is the primary focus of most of the papers included in this review. Studies A, B, C, D, E, F, G, H, I, J, K, L, M, N, O, P, Q, R, S, T, U, Y, and AD present ML models designed to detect impairments in children’s speech. While many of these systems are reliable and could effectively assist therapists in identifying SSDs, most offer only a binary output, detecting whether an abnormality is present without further classification. An exception is study M, which goes beyond detection by analyzing speech after identifying ataxia—early onset ataxia encompasses a heterogeneous group of neurological disorders, either inherited or acquired, typically manifesting before the age of 25^20^—to categorize the severity of the impairment into three levels—a feature that could greatly benefit therapists. However, further work is still needed to develop models that can automatically classify different types of disorders and assess the severity of impairments.

One of the gaps identified in these studies is the limited focus on providing real-time feedback to users, which is a crucial factor in engaging children and encouraging them to continue their practice. Among the papers reviewed, seven studies (F, S, V, W, X, Y, and AD) incorporate some form of feedback, with only two studies offering detailed explanations of their methods. In paper R, they present their SSD detection method, goodness of pronunciation (GOP)—the basic principle of the GOP technique is to measure the ratio between the likelihood of an expected phoneme sequence to the most likely observed phoneme sequence^21^–to provide real-time feedback if any disorder is detected. Paper Y integrates feedback into gameplay, where successful performance allows the game to continue favorably for the player, while incorrect production requires the task to be repeated until improvement is achieved and the desired sound is produced. However, it does not provide further details regarding their audio processing technique.

SLT is a continuous process that progresses gradually. The key factor in this rehabilitation process is tracking progress over time.^22^ Several studies have developed useful components to support this, such as dashboards representing clinical reports and tracking patient progress for therapists. In addition, by creating portals for therapists, children, and parents, these systems enable various users to access tools tailored to their roles: therapists can design and plan SLT activities, while parents and children can follow the progress and tasks assigned. In papers F, W, and AD, various methods and metrics are employed to report progress and monitor tasks completed by patients, allowing therapists to easily analyze the data and create personalized treatment plans.

The main goal of incorporating games and applications in SLT systems, as highlighted in these papers, is to increase children’s engagement with SLT plans. Many studies introduce SLT games; however, based on our inclusion criteria, we focused on games that utilize data processing techniques to analyze user data and assist therapists in some capacity. Papers T, V, W, X, Y, Z, AA, AB, and AC present SLT games and applications. While paper T does not provide detailed information about its games, the other studies describe interactive gameplay that adapts based on the accuracy of the child’s speech tasks. In paper V, an augmented reality (AR) environment is used to simulate daily conversational tasks for the child, who must complete these tasks to progress in the game. Paper W features an avatar as the instructor in a game resembling Domino. These systems employ various approaches to maximize engagement, including customizable gameplay, visual feedback, avatars, and simulations.

**Figure 3.**
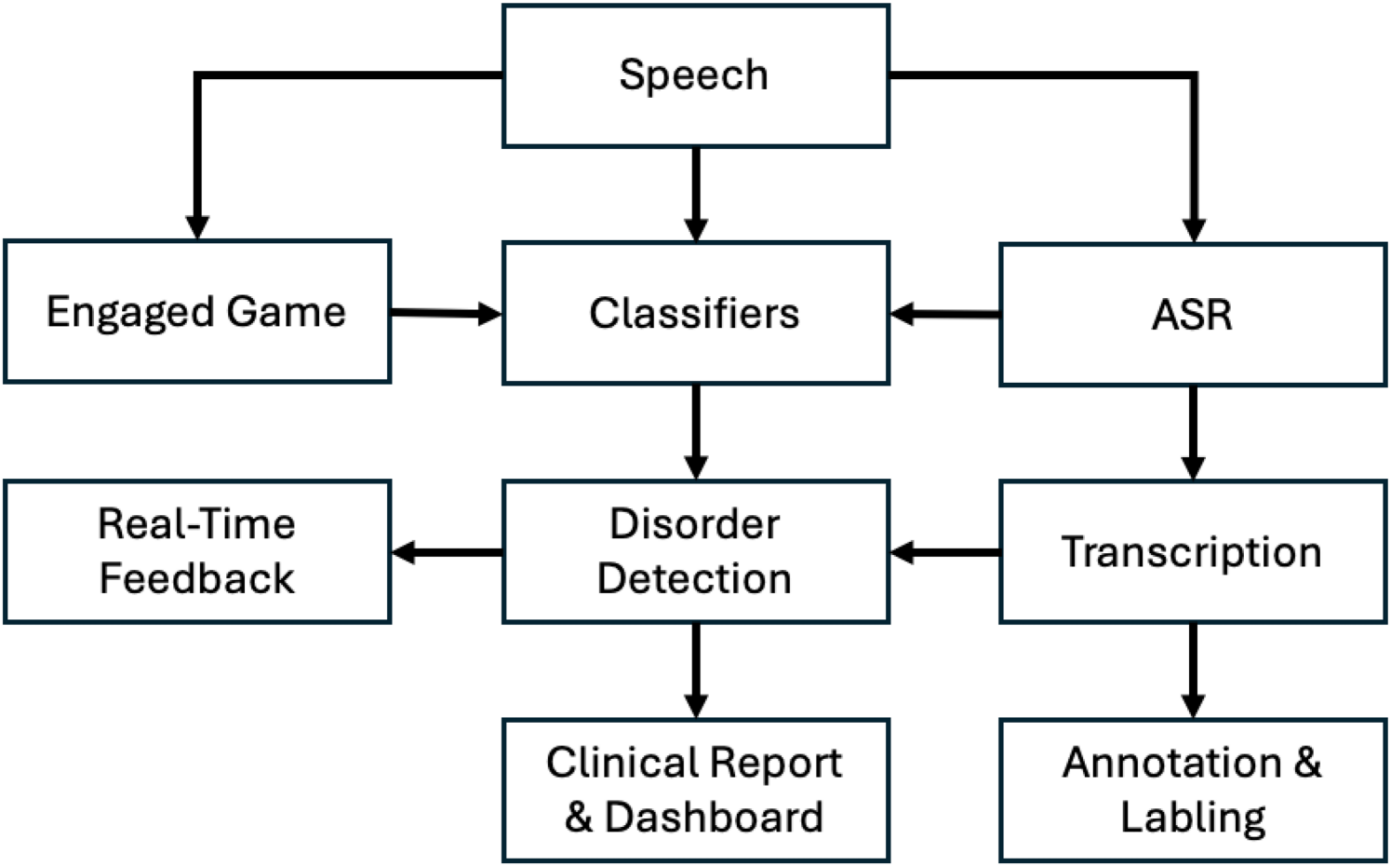
High-level overview of the reviewed approaches, illustrating the key components and their interrelationships.

Table 5 provides a comprehensive overview of the application features presented in the included studies and identifies the papers focusing on each feature. Additionally, Figure 3 illustrates these approaches, highlighting how the components function and their interrelationships.

**Table 5.** The application features provided by these studies.

Application feature	Papers	Overview
Disorder detection	A, B, C, D, E, F, G, H, I, J, K, L, M, N, O, P, Q, R, S, T, U, and Y	All focus on binary detection of an SSD, while F provides a severity classification with three levels.
Real-time feedback	F, S, V, W, X, Y, and AD	Paper S utilizes the GOP technique, Y integrates feedback into gameplay, and paper AD calculates the Levenshtein distance^50^ between the expected pronunciation and the child’s actual pronunciation. The algorithms developed by others are not discussed in detail.
Clinical report generation and dashboards	F, W, and AD	Paper F generates monthly reports, offering insights into the patient’s progress. Paper W provides detailed reports on a child’s progress and history, while paper AD documents the recordings and results of practices completed by the child.
Engaged game	T, V, W, X, Y, Z, AA, AB, and AC	Paper V is an AR environment, paper W features an avatar as the instructor in a game resembling Domino, while others provide regular engaged games.

SSD: speech sound disorder; GOP: goodness of pronunciation; AR: augmented reality.

### AI models and speech processing techniques

In the previous section, we explained the high-level features provided by these systems. Now, we delve into their development processes, focusing on the technical aspects of their work. Since SLT systems are complex and involve multiple technical components cooperating before producing an output, most papers focus on specific parts of this workflow. Currently, there is no comprehensive system that covers all aspects to create a complete SLT application. While some studies focus on initial stages such as data collection and pre-processing, others address later stages such as disorder classification and detection. It is important to recognize that all of these contributions are essential for building a fully functional SLT system.

We identified that almost all the included studies could be categorized into two main approaches: Classification or ASR. Former, primarily involves binary classification of input speech into healthy or SSD, and all the related problems to classification problems. The ASR methods, while they result in detecting impairments as well, they include mainly fine-tuning existing ASR models for adult speech to accommodate children’s speech or introducing new models developed primarily for children’s speech, and also innovations regarding addressing the challenges of data scarcity for training these systems.

Most of the papers in this review introduce speech, audio, and signal processing techniques to varying degrees. One of the main steps is feature selection and extraction to find the most accurate and efficient way for speech representation. The most widely used feature sets in these studies are in papers K, N, O, and P, which are the Geneva Minimalistic Acoustic Parameter Set (GeMAPS) and the extended GeMAPS (eGeMAPS), which consist of 62 and 88 acoustic parameters including the Pitch, Jitter, Formant Frequencies, Shimmer, Loudness, Spectral Energies, first four Mel-Frequency Cepstral Coefficients (MFCCs), and mean and standard deviation of voiced/unvoiced regions, respectively, etc.^51^ While paper C also using eGeMAPS, they mention that computational paralinguistic challenge (ComParE) feature set—A large feature set was introduced in ComParE,^52^ which is well-known for being computationally efficient while effectively representing speech characteristics—outperforms eGeMAPS for SSD detection.

Apart from GeMAPS and eGeMAPS, some studies utilize other acoustic features that, in some cases, overlap with these sets. For instance, papers C, G, R, and Y commonly use MFCCs as one of the main feature sets to feed their models.

In papers I and P, a method called recursive feature elimination (RFE) is used to identify the most efficient set of features. RFE is a supervised feature selection method that works by recursively eliminating the least important features based on the weights assigned by a supervised classifier.^53^ In paper R, they use the forward feature selection algorithm, a method that finds the most effective features in the classification output of the model. These approaches help refine the feature set to achieve the most discriminative acoustic parameters. In paper A, they use the Whisper encoder,^54^ a state-of-the-art ASR model by OpenAI, to extract deep audio features by processing spectrograms through its feature extraction layers and transformer-based encoder. Table 6 summarizes the overall development specifications across the included studies.

**Table 6.** Models and features used in ASR and classification approaches.

Approach	Papers	Model	Papers	Features	Papers
ASR	A, B, E, H, J, L, N, Q, T	CLDNN	N	eGeMAPS	F, N
		CNN	H, L, N	GeMAPS	N
		HMM-DNN	L	71-dimensional Mel-Filterbank features	L
		TDNN	L	MFCC	A, H
		CRDNN	H	Whisper encoder embeddings	A
		GMM-HMM	H	i-Vectors	A
		Whisper	A, B	x-Vectors	A
		ResNet34	A		
		w2v	A, B		
Classification	C, D, G, I, K, M, N, O, P, R, S, U, Y	Bi-GRUs	K	Shimmer	R
		CNN	M	eGeMAPS	P
		Decision tree	O, U	Formant frequency	G, R
		GMM-HMM	C, G	Jitter	R
		HMLM	M	HNR	C, R
		ResNet	O	Fundamental frequency	R
		SVM	A, C, G, I, P, R, S, Y	GeMAPS	K, O, P
		LDA	I	Deep spectrum representation	O
		XGBoost	I	Duration of words and vowels	G
		RF	I	Filter banks	Y
		TDNN	C	i-VECTORS	C, K
		SNN	C	MFCC	C, M, R, Y
		HuBERT	C	PATA spectral centroid	M
		WavLM	C	Posterior features	K
		VGGish	D	Spectral kurtosis	M
				SPI	R
				x-vectors	C, K
				ComParE	C

ASR: Automatic Speech Recognition; GeMAPS: Geneva Minimalistic Acoustic Parameter Set; eGeMAPS: extended GeMAPS; MFCC: Mel-Frequency Cepstral Coefficient; CLDNN: Convolutional Long short-term Memory Deep Neural Network; CNN: Convolutional Neural Network; HMM-DNN: Hidden Markov Model-Deep Neural Network; TDNN: Time Delay Neural Network; CRDNN: Convolutional Recurrent Deep Neural Network; GMM-HMM: Gaussian Mixture Model-Hidden Markov Model; ResNet: Residential Network; w2v: word2vec; Bi-GRU: Bidirectional Gated Recurrent Unit; SVM: Support Vector Machine; HMLM: Hierarchical Machine Learning Model; LDA: Linear Discriminant Analysis; RF: Random Forest; SNN: Siamese Neural Network; HNR: Harmonic-to-Noise Ratio; ComParE: Computational Paralinguistic Challenge; SPI: Soft Phonation Index; XGBoost: eXtreme Gradient Boosting.

The feature selection process is a fundamental step toward achieving accurate and reliable results; however, it is not the only critical step. Model development and classification algorithms combined with the most accurate audio representation approaches are also crucial for success. With countless methods available, selecting the right configuration for a specific task can be a complex challenge. This section of the review focuses on the different classification approaches used in the reviewed papers, identifying the most common methods and analyzing their effectiveness.

Traditional ML algorithms are frequently used in the included studies for classification purposes. Among the various classifiers, support vector machine (SVM) is the most frequently used one as in papers A, C, G, I, K, M, P, R, S, U, and Y, it is employed as the primary classifier. (A SVM is a supervised-learning algorithm that maps data into a high-dimensional space and finds the widest-margin hyperplane that separates classes, relying only on the closest points (the “support vectors”) to define that boundary.^55^) In paper AC, the performance of various ML classification models, such as a radial basis function kernel, random forest, and quadratic discriminant analysis are compared with SVM which in the most cases, SVM consistently outperforms traditional ML algorithms due to its ability to handle high-dimensional feature spaces and its robustness against overfitting in small datasets,^56^ which is a real problem in pediatric SLT.

DNNs and their variants are also being used in certain works for different purposes, such as enhancing ASR models and tuning them for children’s speech, which play a crucial role in mapping raw audio data to phonemes or words—a necessary step for converting the audio data to a more interpretable modality for detecting SSDs. DNNs are effective at learning complex patterns in speech data, eliminating the need for extensive handcrafted feature engineering. However, they present challenges such as higher complexity and the need for larger input datasets.^57^

In paper N, a deep neural network is used for SSD detection, but to address the issue of data scarcity, the problem is reframed as an anomaly detection task. In this approach, the models are trained exclusively on TD speech, which reduces the dependency on disordered speech data. This approach is particularly valuable given the scarcity of annotated corpora for disordered speech. Paper A employs Whisper as its backbone for extracting audio features, first converting input audio into a spectrogram and processing it through 24 encoder blocks. The extracted features are then fed into a multihead classification framework, where age-specific classifiers update only the relevant classification head during training. While it leverages Whisper for feature extraction, its dependency on a fixed ASR reduces its adaptability to SSDs due to their complex patterns. In paper C, the microscopic SSD detection model uses a TDNN-based embedding extractor trained via Siamese neural networks (SNNs) to detect phonological errors in phone-sized speech segments. Errors are identified using cosine distance, which may struggle with subtle articulatory deviations, aggregated, and classified as TD or disordered with an SVM. Paper B uses Whisper as a baseline ASR model without fine-tuning, comparing its performance to a fine-tuned wav2vec2-based model. Wav2vec2 is a self-supervised learning model for speech processing, developed by Facebook AI (Meta). It belongs to the wav2vec 2.0 XLS-R (Cross-Lingual Speech Representation) series, which is designed for multilingual ASR and speech-related tasks. The result shows that the fine-tuned Wav2vec2 outperforms Whisper without additional fine-tuning, which is known for its strong performance and being a breakthrough ASR model in recent years, to highlight the importance of selecting the appropriate model and fine-tuning method for specific clinical purposes. Despite benchmarking Whisper against fine-tuned wav2vec2, this work does not explore the potential benefits of fine-tuning Whisper, which could yield significant improvements.

Paper Q introduces two strategies to overcome the limited availability of large datasets for training ASRs. The first is transfer learning, where a model trained on a large dataset (e.g. LibriSpeech) is fine-tuned on a smaller, specialized dataset (e.g. CSLU Autism Speech Corpus). This technique can also be applied to transfer models trained on adult speech to children’s speech, or from healthy to disordered speech. The second strategy is data augmentation, where additional training data is created by incorporating portions of the OGI Kids’ Corpus, which includes recordings of TD children, to improve the performance of the ASR system.

In summary, while traditional ML models such as SVM remain popular for their effectiveness in smaller datasets, deep learning approaches such as DNNs are increasingly being used, especially in ASR tasks, due to their ability to capture complex patterns. However, data scarcity remains a challenge for DNN-based approaches, prompting the use of techniques such as anomaly detection, transfer learning, and data augmentation to enhance performance.

### Datasets

ML models require large amounts of data to learn and recognize complex patterns between data points, which is crucial for ensuring that systems operate reliably and accurately. As mentioned before, one of the main challenges in working with children’s speech data is the scarcity of available corpora.^12^ A key aspect we aimed to investigate in these papers is the datasets utilized in these studies. While some studies rely on publicly available datasets, others have created their own datasets tailored to specific requirements. It is essential for future research to identify publicly available datasets, leverage those that proved effective in the development process of the included studies, and incorporate insights from works that created their own datasets. This knowledge can help address data-related challenges.

Papers C, D, F, G, H, I, J, K, N, P, Q, S, T, and Y utilize publicly available datasets for their models. The most commonly used dataset is the Ultrasuite Dataset (https://ultrasuite.github.io), which is collected from both TD children and children with SSD. The dataset consists of three subsets: UXTD, which includes speech data from TD children without any signs of speaking difficulty, and two SSD datasets (UXSSD and UPX) containing speech samples from children with various speech disorders, such as childhood apraxia of speech, phonological delay, and articulation disorders.^58^

Papers A, B, E, L, M, O, and R created their own datasets, which consist of speech data from 24 to 709 participants, including both TD children and children with SSDs. In paper E, the authors mention plans to release a dataset containing 400 hours of Mandarin speech data, including 340 hours of adult speech, 30 hours of children reading speech, as well as 30 hours of children’s conversational speech. Table 7 provides a summary of datasets utilized in these papers.

**Table 7.** A summary of the datasets utilized or created by these studies.

Dataset type	Papers	Overview
Public datasets	C, D, F, G, H, I, J, K, N, P, Q, S, T, Y	TIMIT Corpus, AMI Corpus, OGI Kids Corpus, UltraSuite Corpus, CAS Corpus, Corpus of Children’s Pronunciation (CCP), CUChild, LibriSpeech Corpus, CSLU Autism Speech Corpus, PF STAR Dataset, SweSSD Dataset, Ferreira’s Vowel Dataset, CUSENT Corpus, Conti-Ramsden Dataset, ENNI Dataset, Gillam Dataset, Speech Evaluation and Exemplars Database, Swedish Registry dataset, Scandcleft trials, Intercenter study datasets.
Custom-built datasets	A, B, E, L, M, O, R	59 recordings from kindergarten children (healthy and with dysphonia) [R], recordings of 24 Israeli children (healthy and with SSD) [O], single-channel adult speech, child reading and conversational speech [L], a dataset of 55 subjects: 18 healthy children, 21 with progressive ataxia, and 16 with congenital non-progressive ataxia [M], a 4.4-hour child speech dataset with detailed annotations [A], a dataset consisting of speech from 137 participants who uttered all 73 words [B], the speech of 60 children [E].

In the field of automatic speech analysis and assessment, human listeners are still essential for annotation, data labeling, and comparing model outputs with ground truth derived from human feedback. In some of the included studies, human listeners are involved in dataset creation, particularly in transcription tasks. In paper B, human listeners play a crucial role both in transcribing speech and in evaluating the performance of the ASR system. Specifically, three speech-language pathologists transcribed the speech, providing target transcriptions against which the accuracy of the ASR model was measured. Paper E highlights a challenge in transcription accuracy due to the unclear nature of children’s speech, prompting the search for automated solutions to overcome this limitation despite continuing to use human transcribers. In paper O, human listeners annotate the recordings with metadata, including speaker tags, target utterances, and other relevant information about the children, such as their ID, age, and gender, for segmentation and subsequent evaluation. In paper J, human listeners first transcribe the speech and compare it with the ASR model’s output. Then, the results of the SSD detection model are evaluated against human judgments. While these specific studies involve human listeners for several tasks, the remaining studies utilize annotated data from publicly available datasets, some of which are designed for binary classification tasks, specifically identifying whether an impairment is present or absent. Apart from that, papers A, L, M, and R do not explicitly describe their transcription methodologies or clarify the extent to which human listeners were involved in their processes.

## Discussion

The primary goal of this review is to provide researchers with a clear snapshot of the current state of technology in pediatric SLT and serve as a valuable resource for researchers and developers working on developing tools that assist therapists in their sessions or contribute to creating a fully automated SLT system for children with SSD. By offering insights into the models and datasets used in these studies, it seeks to guide future developments in this field.

Throughout the review, various approaches were identified regarding the results and how the authors evaluated their methods. Some papers focused on improving baseline models, such as fine-tuning ASR systems originally designed for adults to accommodate children’s speech, enabling comparisons with existing models. Other studies introduced entirely new approaches that required extensive experimentation to validate their effectiveness. Paper S presents two new GOP techniques—GOP with confidence interval and GOP with SVM—that outperform the baseline GOP technique. Paper N approaches the problem of SSD detection by framing it as an anomaly detection problem. Paper T innovates by constraining the search space of an ASR model to correct phoneme sequences and anticipated articulation errors for the test word. Paper K proposes a unique method by extracting subject-level representations from long utterances, shifting the focus away from individual phonemes or word errors. Paper U tackles the issue by mapping the problem to a situation awareness—“the perception of the elements in the environment within a volume of time and space, the understanding of its meaning and projection of its effects in the near future”^59^—framework, offering a fresh perspective on speech processing. Papers E and I use the landmark analysis (LM) technique, which is an approach that characterizes speech with acoustic markers that are developed based on the LM theory of speech perception.^60–62^ Unlike ASR, LM analysis does not attempt to identify words, but rather to detect acoustic events that occur as the result of sufficiently precise articulatory movements. LM analysis has been suggested as the basis for automatic speech analysis.^63^

The development of SLT systems relies on collaboration between two key fields: technology experts and speech-language therapists. While our review focused on the technological aspects, speech-language therapists apply various intervention methods tailored to specific pathologies,^64^ which is out of our domain of expertise, and we did not evaluate whether these methods are well-aligned with the technology in the reviewed papers. For example, most of the papers focus on the phoneme-level analysis of recorded children’s speech, whereas some papers explore different approaches. In paper T, the focus is on the initial consonants of syllables. Paper G examines the duration of test words with different syllable counts, the duration of the three short vowels /ɐ e o/, and the formant frequencies of the five long vowels /a: i: ɛ: ɔ: u:/. Paper AC uses a “Sustained Vowel" approach, measuring how long a child can produce and maintain a specific vowel sound. In paper M, the standardized clinical PATA speech test from the Scale for the Assessment and Rating of Ataxia^65^ is employed. The PATA test, which is also known as the diadochokinetic task or alternating/sequential motion rates, involves asking the patient to repeatedly say the syllables /pa/, /ta/, and /ka/ in sequence, as quickly and accurately as possible, targeting different parts of the mouth for articulation assessment.

Given that this research pertains to speech, language is a crucial factor. Most of the papers we reviewed focused on English-speaking children, which is understandable given that we primarily examined English-language studies. However, there may be significant efforts and advanced models developed for other languages that were not included in this review, representing a limitation in scope.

Another point worth discussing is the level of detail provided in the papers. While some papers offer comprehensive information, from data collection and pre-processing to model development and justifications for architectural choices, others fall short. Many papers introduce innovative models and approaches, but provide insufficient details on implementation or offer no results to support their findings. In this review, we aimed to balance papers that presented useful approaches with those that provided detailed methodologies.

Regarding the developed systems, common elements among them include pre-processing and feature selection techniques to maximize the efficiency of speech signal processing, experimentation with various configurations of traditional ML classifiers and DNN architectures to develop robust models for SSD detection or classification, efforts to tackle data scarcity, especially in children’s speech, have included approaches such as transfer learning and data augmentation, and development of interactive games and mobile applications to increase user engagement. While notable results have been achieved, there remains a lack of focus on video data, which could play a significant role in this field. A key factor in speech production quality is articulation, which involves the coordinated movement of organs such as the tongue, lips, teeth, and jaw.^66^ Beyond analyzing speech sounds, studying and analyzing articulation video data provides valuable insights into the issues of a child with SSD and enables more accurate patient evaluations. Incorporating video and image processing techniques, along with data synthesis methods, could significantly enhance the current classifiers and represent a major step toward fully automated SLT systems. Although there have been some efforts toward tongue simulation, little has been done to simulate the articulation of SSD speech of children to represent to the therapists. In traditional therapy sessions, therapists closely observe the patient’s articulatory movements to be able to identify the movement issues. Simulating these movements digitally could be a valuable tool in automated systems.

Data scarcity is one of the most significant challenges highlighted by nearly all the papers in this review. ML models require substantial amounts of data to deliver reliable results,^67^ yet the lack of large, annotated datasets remains a persistent issue. Another commonly mentioned challenge is the low quality of annotated data for children, primarily due to the wide variability in pronunciations among younger speakers.^12^ Moreover, data privacy concerns, particularly related to children’s speech, have not been adequately addressed in these studies. While large datasets are crucial for achieving reliable and accurate systems, their collection is hindered by strict privacy regulations. Potential solutions include leveraging simulation models, digital twins, or advanced data anonymization techniques. Regarding the current state of SLT technologies, many existing systems, such as ASRs, are predominantly designed for adult speech, with limited efforts focused on children’s speech. This creates additional challenges when addressing speech disorders in younger populations. Another notable limitation is that current systems often detect impairments but fail to specify the severity or type of SSD. This differentiation is essential for tailoring rehabilitation plans to the specific needs of the child and their unique type of SSD.

Furthermore, therapist supervision remains essential in the SLT journey for children. While significant efforts have been made toward automating SLT systems, they have only been partially successful in addressing the challenges. Studies A, B, C, D, E, F, G, H, I, J, K, L, M, N, O, P, Q, R, S, T, and U focus on the diagnosis of SSDs but do not offer engaging, game-like experiences or design personalized practices based on individual impairments and progress. As a result, therapist supervision is still necessary for planning and managing a child’s progress. On the other hand, studies V, W, X, Y, Z, AA, AB, AC, and AD have developed games and applications, some of which provide real-time feedback. However, they still lack automatic practice design tailored to the child’s progress or a detailed analysis of the causes and symptoms of SSDs. These tools excel at generating reports for therapists, reducing their workload, and freeing up more time for other patients. Although not fully automated, they offer valuable support to therapists by easing their workload.

In the present study, while we aimed to provide a comprehensive and practical snapshot of the field, several limitations must be noted. Firstly, as previously mentioned, our primary focus is on technological aspects, specifically AI. However, this field is inherently multidisciplinary, involving both speech pathology experts and technology developers. Therefore, evaluating the pathological components and considering alternative approaches and their alignment with current AI advancements remains necessary. Additionally, our review was restricted to English-language articles, potentially overlooking relevant research published in other languages. Given the rapid advancement of AI in this domain, we chose to concentrate primarily on papers published after 2018. This decision enabled us to better capture the current state-of-the-art models and methodologies, providing a more focused basis for future developments. Furthermore, our search strategy utilized three keyword groups, one explicitly related to “children," as child speech models significantly differ from those designed for adults. Consequently, this method might have excluded some studies not explicitly mentioning children in their titles, yet still relevant to the domain. Finally, our scope was intentionally narrowed to AI-based systems due to our specific area of expertise, excluding potentially useful studies employing alternative technological approaches.

While technologies such as acoustic feature extraction, signal processing algorithms, and classifiers have been thoroughly explored, many crucial aspects discussed in this section remain underexplored. These gaps must be addressed to create fully automated, reliable, and comprehensive SLT systems that the field needs.

## Conclusion

In this scoping review, we highlight the main advancements in AI in pediatric SLT systems, as presented in the current literature. We aimed to identify the state-of-the-art approaches presented in these studies and investigate the challenges and obstacles faced by researchers in this field. Significant progress is evident in ML algorithms for SSD detection, the adaptation of ASR systems for children’s speech, and the development of interactive applications, all demonstrating AI’s potential to address limited access to SLTs and the high demand for therapy services. Evaluations across these studies show that SLT systems are effective in engaging children in practicing SLT activities, making these systems a valuable asset in the field. However, our findings reveal notable limitations still needing resolution: the lack of a large, accurately annotated corpus, binary SSD classification without severity grading and the type of SSD classification, the need for more efforts toward creating child-specific models, a lack of multimodal systems and use of video data and video processing for articulation analysis, and single-language applications. Each of these studies makes steps toward a fully integrated SLT system for children, though combining this knowledge is essential for a comprehensive solution. We hope this review serves as a foundation for future research, equipping developers with a broad understanding of the field and facilitating advancements toward a complete AI-driven SLT system.
